# Development of Machine Learning Algorithms for the Prediction of Financial Toxicity in Localized Breast Cancer Following Surgical Treatment

**DOI:** 10.1200/CCI.20.00088

**Published:** 2021-03-25

**Authors:** Chris Sidey-Gibbons, André Pfob, Malke Asaad, Stefanos Boukovalas, Yu-Li Lin, Jesse Creed Selber, Charles E. Butler, Anaeze Chidiebele Offodile

**Affiliations:** ^1^Department of Symptom Research, University of Texas MD Anderson Cancer Center, Houston, TX; ^2^Department of Obstetrics and Gynecology, Heidelberg University, Heidelberg, Germany; ^3^Department of Plastic Surgery, University of Texas MD Anderson Cancer Center, Houston, TX; ^4^Department of Health Services Research, University of Texas MD Anderson Cancer Center, Houston, TX; ^5^Institute for Cancer Care Innovation, University of Texas MD Anderson Cancer Center, Houston, TX; ^6^Baker Institute for Public Policy, Rice University, Houston, TX

## Abstract

Financial burden caused by cancer treatment is associated with material loss, distress, and poorer outcomes. Financial resources exist to support patients but identification of need is difficult. We sought to develop and test a tool to accurately predict an individual's risk of financial toxicity based on clinical, demographic, and patient-reported data prior to initiation of breast cancer treatment.

**PATIENTS AND METHODS:**

We surveyed 611 patients undergoing breast cancer therapy at MD Anderson Cancer Center. We collected data using the validated COmprehensive Score for financial Toxicity (COST) patient-reported outcome measure alongside other financial indicators (credit score, income, and insurance status). We also collected clinical and perioperative data. We trained and tested an ensemble of machine learning (ML) algorithms (neural network, regularized linear model, support vector machines, and a classification tree) to predict financial toxicity. Data were randomly partitioned into training and test samples (2:1 ratio). Predictive performance was assessed using area-under-the-receiver-operating-characteristics-curve (AUROC), accuracy, sensitivity, and specificity.

**RESULTS:**

In our test sample (N = 203), 48 of 203 women (23.6%) reported significant financial burden. The algorithm ensemble performed well to predict financial burden with an AUROC of 0.85, accuracy of 0.82, sensitivity of 0.85, and specificity of 0.81. Key clinical predictors of financial burden from the linear model were neoadjuvant therapy (β_regularized_, .11) and autologous, rather than implant-based, reconstruction (β_regularized_, .06). Notably, radiation and clinical tumor stage had no effect on financial burden.

**CONCLUSION:**

ML models accurately predicted financial toxicity related to breast cancer treatment. These predictions may inform decision making and care planning to avoid financial distress during cancer treatment or enable targeted financial support. Further research is warranted to validate this tool and assess applicability for other types of cancer.

## INTRODUCTION

Over the past decade, increasing public and policy attention has been paid to the issue of high treatment-associated costs for cancer. Cancer is currently the second most expensive chronic condition with an annual outlay estimated to be $157 billion in US dollars in 2020.^1^ The term financial toxicity denotes the deleterious effects of cancer treatment costs on the subjective and material experience of patients with cancer.^2,3^ Financial toxicity has been associated with worsening oncologic, psychosocial, and economic outcomes across several cancer types.^3-7^ According to recent estimates, 48%-73% of cancer survivors experience some ill effects of financial toxicity.^5^

**CONTEXT**

**Key Objective**
Can machine learning (ML) algorithms predict treatment-associated financial toxicity in patients with breast cancer who undergo surgical treatment by using clinical, demographic, and patient-reported data?
**Knowledge Generated**
The algorithm performed well to predict financial burden with an area-under-the-receiver-operating-characteristics-curve of 0.85 and a sensitivity of 0.85. Key clinical predictors of financial burden were neoadjuvant chemotherapy and autologous rather than implant-based breast reconstruction.
**Relevance**
ML models accurately predicted financial toxicity related to breast cancer treatment. These predictions may inform decision making and care planning to mitigate financial distress during cancer treatment or enable targeted financial support.

Breast cancer has become the representative condition for financial toxicity in cancer owing to its annual incidence, public health awareness, and policy relevance.^8,9^ Although the etiology is multifactorial, there is limited evidence surrounding the patient- and treatment-level risk factors associated with financial toxicity among breast cancer surgical patients. Of note, much of the existing literature on financial toxicity is centered on the medical oncology experience.^3^ This is salient because over 60% of index breast cancer presentations are early-stage and therefore amenable to surgical treatment.^10^

The 11-item COmprehensive Score for financial Toxicity (COST) questionnaire is a cancer-specific, prospectively validated patient-reported outcome measure (PROM) that provides insights into the manifestations of treatment costs-associated financial distress.^11,12^ Precise identification of at-risk population segments may enable targeted financial support. However, an accurate prediction of which individual patient will experience financial toxicity is inherently challenging. This is because financial toxicity is a complex interplay of patient demographic, disease level, and treatment-related factors. Machine learning (ML) may help clinicians and health systems overcome this challenge. ML is a branch of artificial intelligence that leverages automated mathematical model creation to iteratively learn from input data, resulting in the identification and/or prediction of a future state.^13-16^ ML algorithms may help identify which complex combination of patient and treatment characteristics make it more likely to experience financial toxicity by comparing those patients with financial toxicity to those without financial toxicity during the training process of the algorithm. Less complex ML algorithms (eg, regularized regression) are readily interpretable but their performance is limited to identifying linear intervariable relations; more complex ML algorithms (eg, classification trees, support vector machines [SVMs], and neural networks) can identify nonlinear intervariable relations but are less comprehensible.^14,16^ In the present article, we explore whether supervised ML algorithms can reliably predict financial toxicity in patients with breast cancer who undergo surgical treatment. These predictions may inform decision making and care planning to avoid financial distress during cancer treatment or enable targeted financial support for individuals.

## PATIENTS AND METHODS

### Data Source

The University of Texas, MD Anderson Cancer Center's Institutional Review Board approved the present study. This is a single-institution cross-sectional survey of consecutive adult female patients who underwent lumpectomy or mastectomy (skin-sparing, total, nipple-sparing, or modified radical) over an 18-month period (January 2018-June 2019). All human participants provided written informed consent. All eligible procedures were performed under the indication of cancer or risk reduction (ie, BRCA mutation).

Inclusion criteria were age > 18 years and the ability to comprehend our survey in English. Our electronic survey, with an embedded consent form, was deployed to the patient population (N = 2,293) on June 2019. Nonresponses triggered three email reminders. The survey comprised the COST questionnaire and a purpose-built 30-item questionnaire that elicited patient-reported information on several domains shown to be relevant to financial hardship in cancer.^3^ The COST questionnaire is well represented in the health services research literature and has been described previously.^11,12,17-20^ A total of 2,293 patients participated in the survey; 611 patients (26.6%) completed the survey and were used in our analytical sample.

### Candidate Covariates

Table 1 outlines the baseline demographic information and covariates related to treatment course that were abstracted from the purpose-built survey and attendant electronic medical record (EMR), respectively. These patient- and treatment-level factors were selected for their ability to engender the construction of a prediction tool to facilitate pre-operative risk facilitation for financial toxicity.

**TABLE 1.**
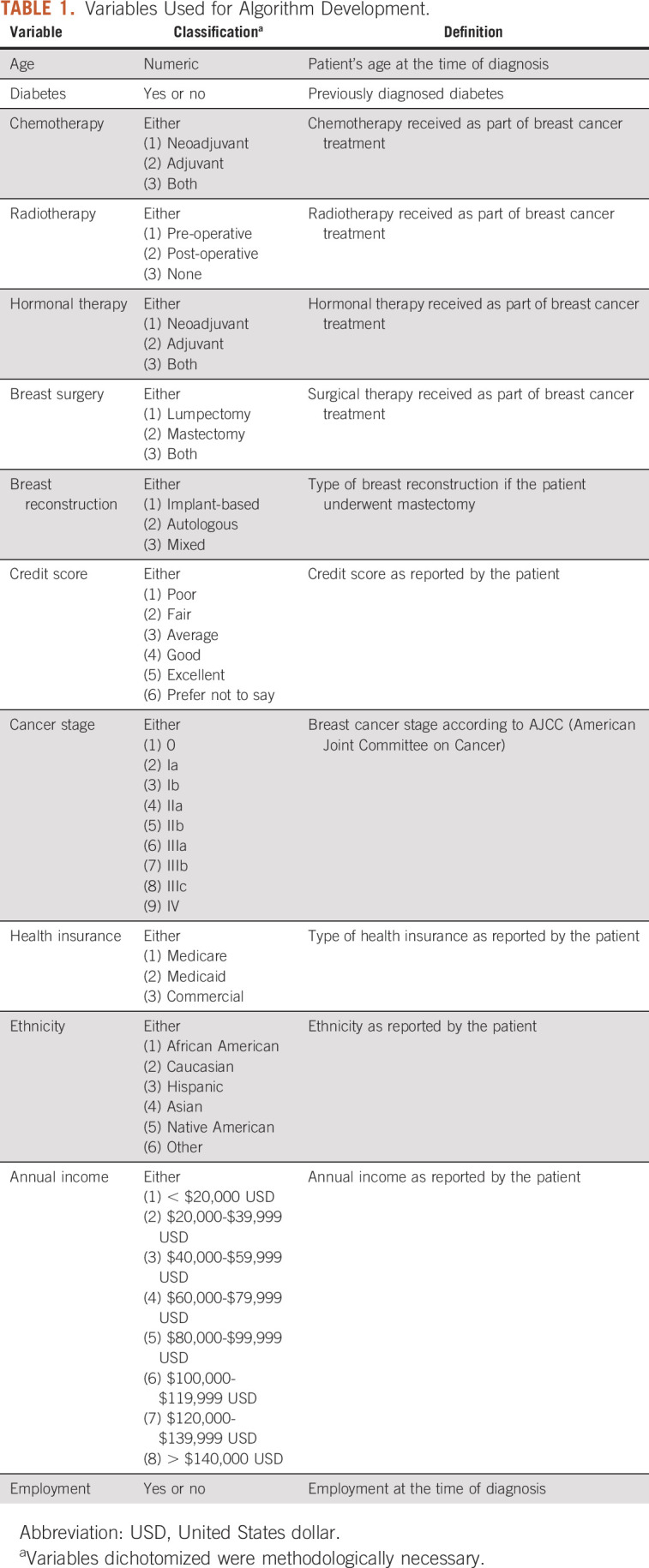
Variables Used for Algorithm Development.

### Missing Data

Missing data were computed using multivariate imputation by chained equations.^21^ There were 1% of missing values in the data set—there were no missing values in the outcome variable.

### Outcome

The primary outcome was the ensemble's ability to accurately identify women who experienced high financial burden. We defined financial burden as a COST score ≤ 23. Briefly, the COST questionnaire is scored from 0 to 44, and lower values represent greater treatment-induced financial distress. This cutoff was both commensurate with (1) previous research using the measure^11,17^ and (2) an average COST score of 23 for those women who responded that they experienced significant financial burden on a single-item question we added to the questionnaire. Finally, this cutoff engendered a more robust analysis as the median COST score for our population was 28.

### Statistical Analysis

The development of our algorithms was informed by guidelines on how to use ML in medicine^13^ and diagnostic tests^22^ as well as previously published research by our group.^16,23,24^ We provide a detailed description of the algorithm development according to these guidelines in the Data Supplement. We used the Prediction Model Study Risk of Bias Assessment Tool (PROBAST) to ensure high accuracy when these models were used elsewhere.^25^ We normalized the range of all values using feature scaling. We then trained and tested four ML algorithms (neural network, regularized regression, SVMs, and regression trees) using the open-source R statistical programming software (RStudio, Boston, MA). Four algorithms were selected based on their demonstrated high performance on similar predictive tasks.^23,24^ Algorithms are introduced briefly below, and details on the algorithm development are published elsewhere and in the Data Supplement.^16^

#### Regularized regression using the elastic net.

Regularized logistic regression is a type of regression where the regularization process effectively selects features to include in the model ensuring that the model has a reduced risk of bias and increased likelihood of generalizability to new data sets. Aside from improved generalizability, the regularized regression provides coefficients that can be interpreted to understand why the model is making the decisions it is making (Table 4).^14,16^

#### Classification tree.

Classification trees are algorithms that use recursive partitioning of data to fit a simple prediction model with a single rule (eg, is body mass index [BMI] > 30) at each partition node (Fig 2). The tree comprised multiple decision nodes that guide the algorithm toward the correct classification. Classification trees are capable of providing both a clearly interpretable decision-making process as well as modeling more complex nonlinear relationships between predictors and outcomes, which is why we have selected this algorithm for our analysis to predict financial toxicity.^14,16^

#### Support vector machines.

SVMs are binary classifier algorithms that seek to create a linear boundary that separates classes in a high-dimensional feature space. To successfully create linear separators (known as hyperplanes) within complex nonlinear data sets, SVMs use a technique known as a kernel trick. The kernel trick allows the algorithm to transform the input data to straighten out the complexity and to allow a linear hyperplane to separate the classes (eg, will a patient experience financial toxicity or not).^14,16^

#### Neural network.

Neural networks are complex algorithms inspired by the structure of the human brain and consist of connected units (neurons). Similar to the human brain, neural networks operate by sending signals throughout their architecture, which are combined and modified at each neuron before proceeding through the entire architecture of the network and, eventually, leading to a decision. Neural networks can process large amounts of unstructured data and identify the most distinct patterns—as we do not know all possible influencing variables yet in the prediction of financial toxicity, we also used this algorithm for our analysis.^14,16^

We combined predictions from each of the individual algorithms into a voting ensemble to derive final predictions of financial burden.^16^ Data were randomly partitioned into training and test samples with a 2:1 ratio. Metrics used for model performance assessment were overall accuracy, sensitivity (correctly identified women who would experience financial toxicity), specificity (correctly identified women who would not experience financial toxicity), and area-under-the-receiver-operating-characteristics-curve (AUROC).

## RESULTS

### Patients

The 611 patients in the full analysis set were randomly partitioned (2:1 ratio) into a training set for the algorithm development (n = 408) and a test set (n = 203). The mean age at baseline was 58 ± 12 years and BMI was 28.7 ± 6.5. Caucasians were the predominant racial category (76.8%), and 60% of patients reported an annual income > $80,000 in US dollars. The most common clinical tumor category was stage I in 273 women (44.7%), and most women (55.5%) did not receive chemotherapy (neoadjuvant or adjuvant). Fifty-seven percent of patients underwent lumpectomy and 61% of patients received adjuvant hormonal therapy. Regarding the effect of financial toxicity on patients, 367 (40.1%) at least once decreased their basic spending on things such as food and clothing; 412 (67.5%) at least once decreased their spending on leisure activities such as vacations, eating out, or movies; 38 (6.2%) delayed breast surgery because of concern about costs; and 75 (12.3%) skipped a clinic visit (oncology, breast surgery, and plastic surgery) to save on costs. Complete baseline demographic and clinical characteristics are outlined in Table 2.

**TABLE 2.**
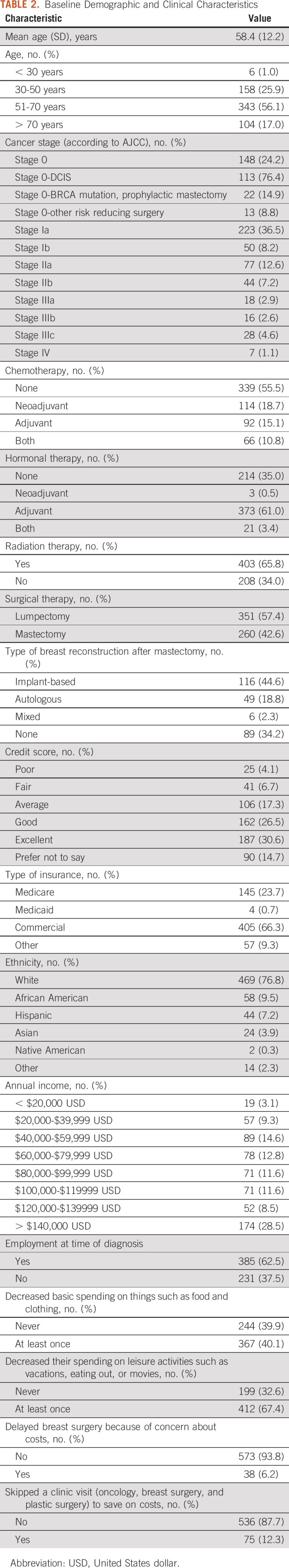
Baseline Demographic and Clinical Characteristics

### Algorithm Performance

**FIG 1.**
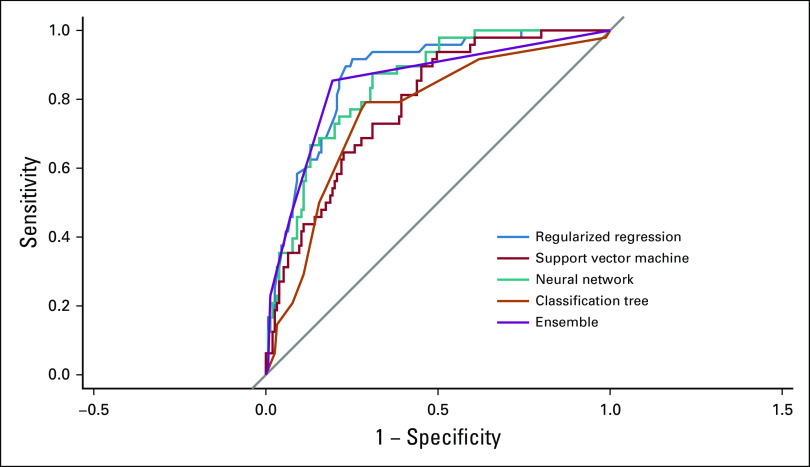
Receiver operating characteristics curves for the machine learning algorithms.

When applied to baseline data, the algorithm reliably predicted women who would later experience financial burden because of treatment (Table 3). In the test set of 203 women, the overall accuracy of the algorithm ensemble was 0.82 (95% CI, 0.76 to 0.87). The algorithm ensemble correctly identified 41 of 48 women who later experienced financial burden (sensitivity = 0.85; 95% CI, 0.72 to 0.94), and 125 of 155 without a financial burden (specificity = 0.81; 95% CI, 0.74 to 0.87). Combining the predictions of the four single algorithms into a voting ensemble improved sensitivity to predict financial toxicity. Of the single algorithms, the neural network showed highest sensitivity (64.6%; 95% CI 49.5 to 77.8). The area under the ROC curve for the algorithm ensemble was 0.85 (95% CI, 0.79 to 0.91). ROC curves are shown in Figure 1.

**TABLE 3.**
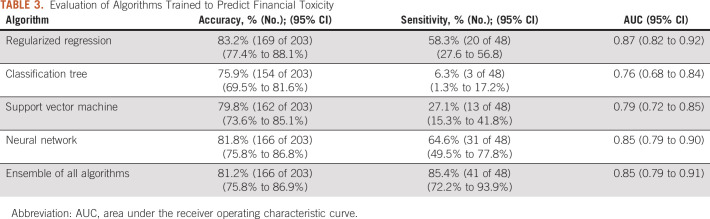
Evaluation of Algorithms Trained to Predict Financial Toxicity

### Factors Associated with Financial Burden

**FIG 2.**
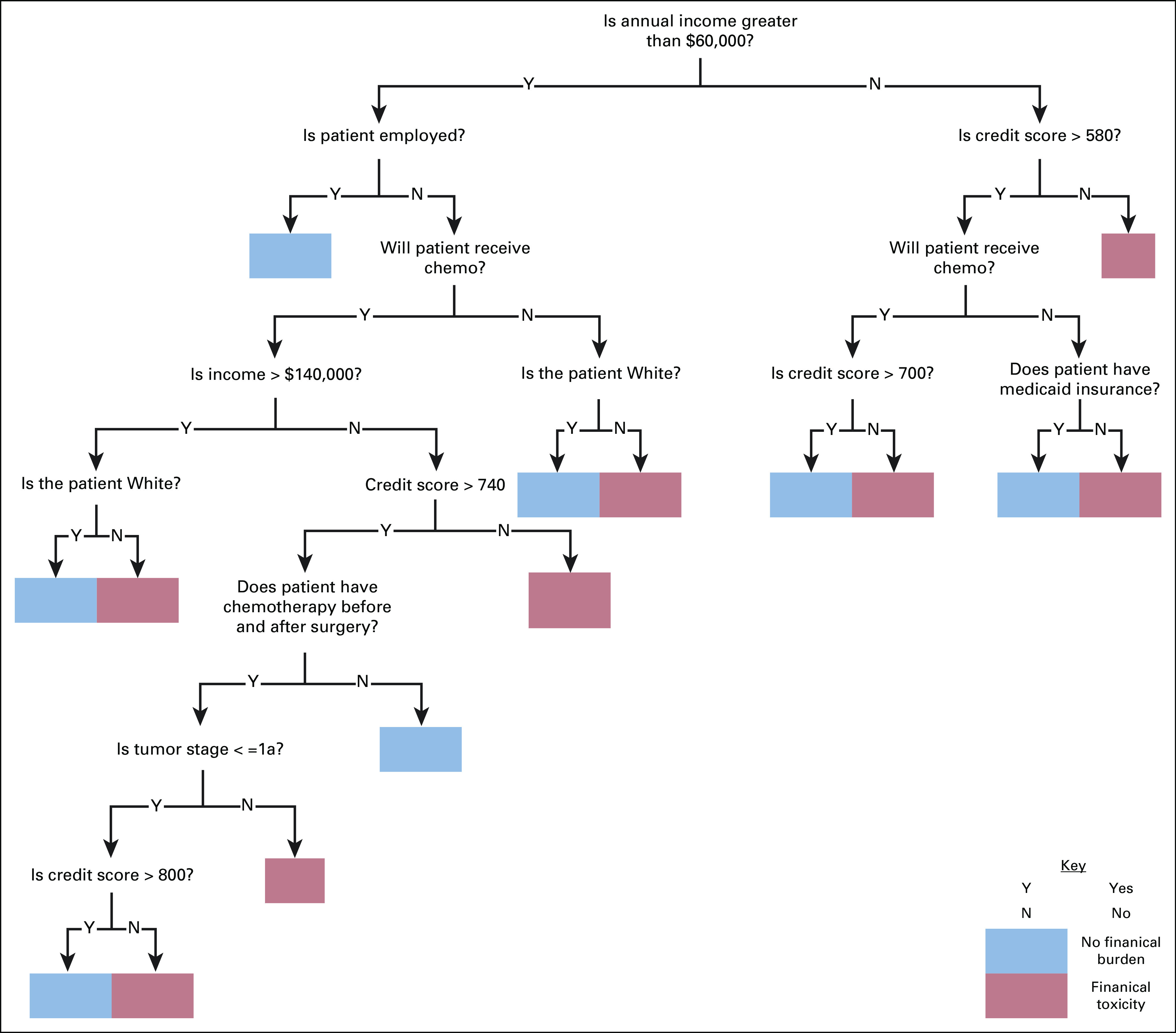
Classification tree structure.

We analyzed the coefficients in the regularized regression model (Table 4) and the classification tree. The following were identified as being key clinical predictors of financial burden in both models: neoadjuvant therapy (β_regularized_, .11) and low credit score (β_regularized_, .22). With respect to the latter, an inverse relationship between worsening credit score and risk of financial burden was also appreciated, that is, poor credit score (β_regularized_, .22), fair (β_regularized_, .05), average (β_regularized_, .05), good (β_regularized_, −.02), and excellent (β_regularized_, −.09). Both Medicare and Medicaid insurance lowered the risk of financial burden and African Americans were noted to be at higher risk to experience financial burden (β_regularized_, .01) relative to Caucasians (β_regularized_, .00). In the regularized regression model, the receipt of autologous reconstruction was a key predictor of financial toxicity (β_regularized_, .06). Notably, radiation and clinical tumor stage had no apparent effect on financial burden in either model. The classification tree is shown in Figure 2.

**TABLE 4.**
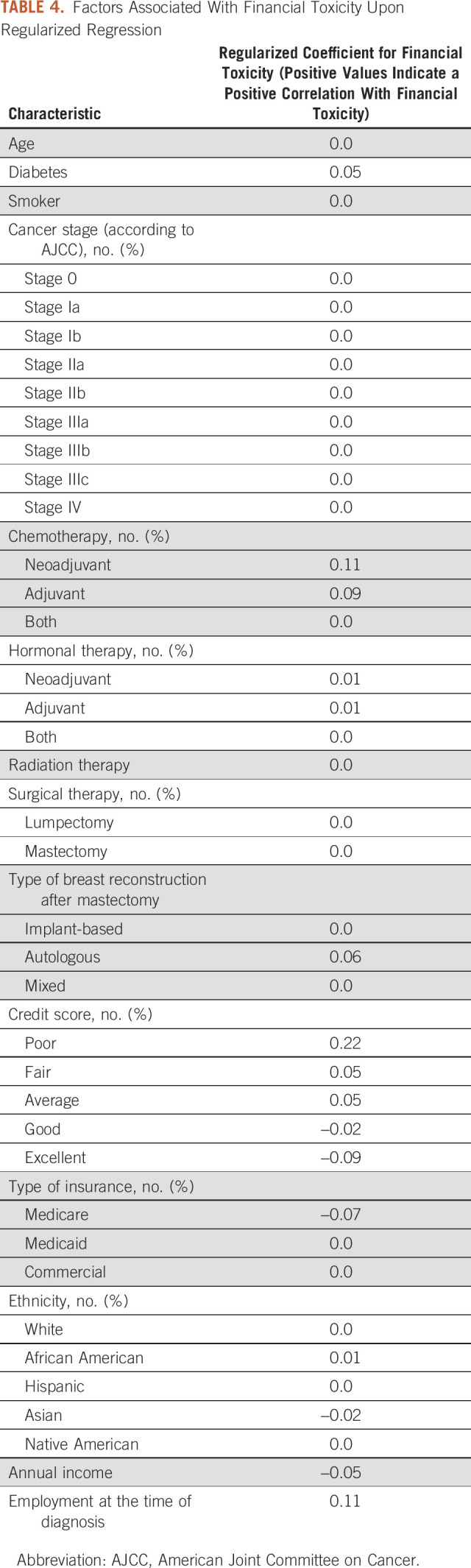
Factors Associated With Financial Toxicity Upon Regularized Regression

### Analysis of Misclassified Patients

There were seven cases (14.6%) where the algorithm incorrectly predicted that financial burden would not be experienced when in fact it was. The Data Supplement compares the characteristics of the seven misclassified patients to the whole cohort. These patients had a lower cost score (15.6 *v* 28.0), a higher incidence of major complications (28.6% *v* 7.5%), were less likely to be employed (57.1% *v* 62.5%), and younger (mean age 53 years *v* 58 years).

## DISCUSSION

In the present study, we demonstrate that the occurrence of financial toxicity can be accurately predicted prior to the initiation of treatment using ML algorithms trained on patient-reported, perioperative, and clinical data. Evaluation of algorithms suggested that receipt of neoadjuvant chemotherapy, utilization of autologous breast reconstruction, and a low credit score as being significantly associated with a risk of financial toxicity. Our supervised learning algorithm demonstrated good discriminatory performance with an AUROC of 0.85, accuracy of 0.82, sensitivity of 0.85, and specificity of 0.81. Our intent with the present study was to establish a methodologic framework for (1) making financial toxicity actionable (ie, target for interventions) and (2) embedding financial toxicity assessment into clinical workflows (eg, artificial intelligence algorithms integrated into EMRs can flag high-risk patients at the time of pre-operative appointments).

It is clear that advances in breast cancer treatment have yielded measurable gains in clinical outcomes for these patients.^10^ Unfortunately, they have also led to undue financial burden for patients, their families, and the larger society. Financial toxicity is a well-described sequelae of contemporary cancer treatment that has been shown to incite the following adverse effects: worse health-related quality of life,^2,26^ treatment nonadherence,^27^ employment disruption,^28^ bankruptcy,^4^ early mortality,^29^ and maladaptive behaviors.^27^ Examples of maladaptive behaviors intended to lower costs include skipping clinic visits, reducing medication doses, and adjustments in nonmedical spending (travel, food, education, and clothing).^2,3,17^ Recent evidence has also shown that women of lower socioeconomic status prioritize treatment costs over certain aspects of their cancer care such as receipt of reconstruction.^30,31^

Strategies to mitigate financial toxicity include provider education, deployment of financial navigators, and increasing access to copay assistance and charity care programs.^3^ In the absence of a financial toxicity prediction algorithm to appropriately subsegment or identify high-risk patients with breast cancer, the expense and indirect costs of implementing these mitigation strategies at scale (ie, deployed to every patient with breast cancer) would be prohibitive. Thus, ML algorithms may be a powerful instrument that reliably predicts the future experience of financial toxicity: it would allow the care team to (a) subsegment patients with breast cancer into high-risk populations for financial toxicity based on *predicted* COST score, (b) tailor the deployment of financial toxicity mitigation tools to this population, for example, financial navigators, charity aid, or insurance copay assistance programs, and (c) guide shared decision making with respect to high-risk patient populations to align treatment decisions with their preferences and values. Additionally, accurate predictions of financial toxicity may also guide the development and implementation of personalized treatment strategies concordant with the values and healthcare spending preferences of patients.^32^

Given the immense societal and policy salience of financial toxicity, risk-factor identification among surgical patients has become an area of considerable research focus.^18,30,33,34^ Our aforementioned results that identified neoadjuvant chemotherapy and poor credit as significant financial toxicity risk factors are not unexpected and in line with the extant literature. A 2019 systematic review by Lee et al. identified chemotherapy as an independent risk factor for financial toxicity, whereas radiation therapy and ablative surgery were not consistently associated with increased risk.^35^

In a 2018 study by Dean et al., poor self-reported credit quality was identified as a marker of economic burden, perceived stress, and worsening overall mental and physical health in breast cancer survivors.^36^ Our work expands on this association through the use of a validated instrument (ie, COST score), a robust analytical approach, and larger study cohort. More importantly, it draws attention to the possible utility of credit score surveillance as a screening tool for early referral to financial counselors and social work.^3^

Our finding that autologous reconstruction is associated with an increased risk of financial toxicity compared with implant-based reconstruction warrants further investigation in light of evidence of increased utilization rates in the United States.^37^ Breast reconstruction decisions are highly preference-sensitive in relation to both the indication and subtype pursued. Financial toxicity, therefore, introduces a new patient-borne dimension to the evaluation of these two healthcare interventions. Prospective studies are needed to (a) validate this finding and (b) query its influence on the decision to undergo autologous breast reconstruction.

Our study is subject to several limitations that warrant mention. First, it was performed at a single, quaternary care institution that may not be representative of other patients with breast cancer or oncology practices. Prospective, multicenter studies involving diverse populations will be needed to verify the validity and generalizability of our algorithm prior to any consideration for implementation into clinical workflows.^13^ Second, the cross-sectional nature of our survey introduces variability in data completeness and limits the veracity of our analysis. Third, the survey components that are dependent on self-reported data might generate some imprecision in our estimates. Fourth, although we agree that a prediction accuracy of 82% is not optimal, it still represents a marked improvement over the current standard of care, which is an unstructured and nonsystematic approach (ie, clinician intuition) of assessing the risk of financial toxicity in patients with breast cancer. Furthermore, it is performed in a retrospective fashion (ie, after the end of treatment) that dampens the utility and overall effectiveness of reducing financial toxicity in the first place (see above). However, our algorithm failed to predict financial toxicity in seven (15%) cases. In-person analysis (Data Supplement) revealed that these patients had a higher incidence of major complications (28.6% *v* 7.5%) likely driven by a higher predisposition toward obesity and diabetes. Complications have been shown in prior studies to result in significant personal financial burden after cancer surgery.^3,38^ Additionally, our misclassified patient segment was less likely to be employed (57.1% *v* 62.5%) at the time of diagnosis. A synergistic effect between these two risk factors (unemployment and increased complications) for financial toxicity, identified in other studies,^3^ may have led to their misclassification. Predictive performance of our algorithm may have also limited by unmeasured confounders. More detailed information (eg, about the molecular subtype of breast cancer or radiation therapy) may allow for more accurate predictions of the model. Future prospective, multicenter research is indicated to overcome this limitation. Moreover, qualitative approaches among the patients who were misclassified by our algorithm may help to identify additional relevant variables in the prediction of financial toxicity. Fifth, as there is no conceptual framework for financial toxicity grading yet, our algorithm does not incorporate different financial toxicity grades. We hope to address financial toxicity grades in future model iterations. Sixth, our survey response rate of 28% can also lead to selection bias because of systematic differences between responders and nonresponders. However, we observed a good distribution of COST scores (financial toxicity) among these 28% who responded to the survey, which minimizes possible response bias. Seventh, the discriminative performance of the single four algorithms was heterogenous with AUC values ranging from 0.76 (95% CI, 0.68 to 0.84) for the regression tree algorithm and 0.87 (95% CI, 0.82 to 0.92) for the regularized regression algorithm. Sensitivity to predict financial toxicity ranged from 6.3% (95% CI, 1.3% to 17.2%) for the regression tree to 64.6% (49.5% to 77.8%) for the neural network. Combining the single algorithm into a voting ensemble improved the prediction of financial toxicity. This is an interesting finding as it implies that different ML algorithms actually identified different patterns for financial toxicity among the same patient group and the combination into a voting ensemble has further improved this pattern identification.

Several strengths can also be identified in the present study. First, to the best of our knowledge, ours is the first report in the literature of the deployment of an ML algorithm for financial toxicity prediction. Additionally, our use of a condition-specific PROM (ie, COST tool) and discriminative algorithms facilitate robustness in our results.

In conclusion, ML provides a set of powerful tools that have the potential to facilitate individualized data-driven, shared decision making in breast cancer care.^23^ ASCO has already asserted that financial considerations be included in the cancer treatment planning process.^39,40^ This is most germane in early-stage breast cancer wherein there are multiple surgical treatment options with comparable efficacy but different cost profiles.^41^ ML techniques can be used to develop a clinical decision-support aid for patients undergoing mastectomy or breast conservation therapy. This allows for (a) optimization of peri-operative counseling, (b) timely referral to financial navigators, and (c) a more patient-centered delivery paradigm. Our research provides evidence that ML models applied to baseline data can accurately predict the future experience of financial toxicity related to breast cancer treatment. Considering the rising costs of cancer care, the potential for individualized data-driven predictions of financial burden holds enormous promise for the creation of a learning health system in oncology.
